# Supplementary material for: Highly Thermal Conductive and Electrical Insulating Epoxy Composites with a Three-Dimensional Filler Network by Sintering Silver Nanowires on Aluminum Nitride Surface
Source: Polymers (Basel). 2021 Feb 25;13(5):694. doi: 10.3390/polym13050694 (PMC7956527; doi:10.3390/polym13050694)
Supplement: Supplementary file 1 [file polymers-13-00694-s001.pdf]

polymers

MDPI

Supplementary Materials

Highly Thermal Conductive and Electrical Insulating Epoxy Composites with a Three-Dimensional Filler Network by Sintering Silver Nanowires on Aluminum Nitride Surface

Wondu Lee and Jooheon Kim *

School of Chemical Engineering & Materials Science, Chung-Ang University, Seoul 156-756, South Korea; sems212@naver.com

* Correspondence: jooheonkim@cau.ac.kr

Citation: Lee, W.; Kim, J. Highly Thermal Conductive and Electrical Insulating Epoxy Composites with a Three-Dimensional Filler Network by Sintering Silver Nanowires on Aluminum Nitride Surface. *Polymers* **2021**, *13*, 694. https://doi.org/10.3390/polym13050694

Academic Editor Angels Serra

Received: 9 February 2021
Accepted: 21 February 2021
Published: date

Publisher's Note: MDPI stays neutral with regard to jurisdictional claims in published maps and institutional affiliations.

Copyright: © 2021 by the authors. Submitted for possible open access publication under the terms and conditions of the Creative Commons Attribution (CC BY) license (http://creativecommons.org/licenses/by/4.0/).

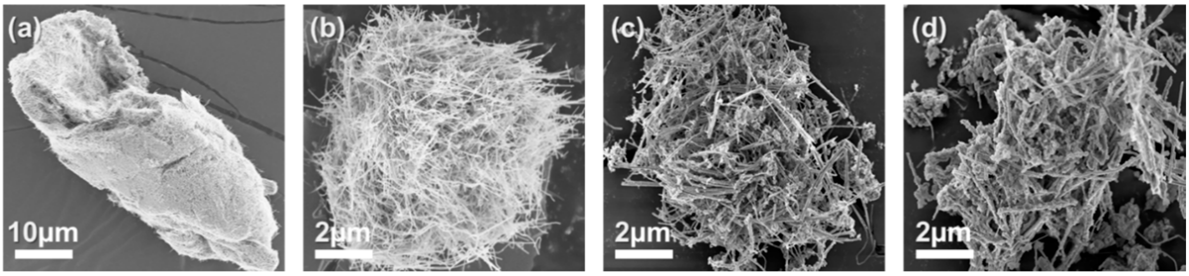

Figure S1. FE-SEM images of AA filler along AlN-to-AgNW ratio (**a**) 1:2, (**b**) 1:1, (**c**) 2:1, (**d**) 3:1.

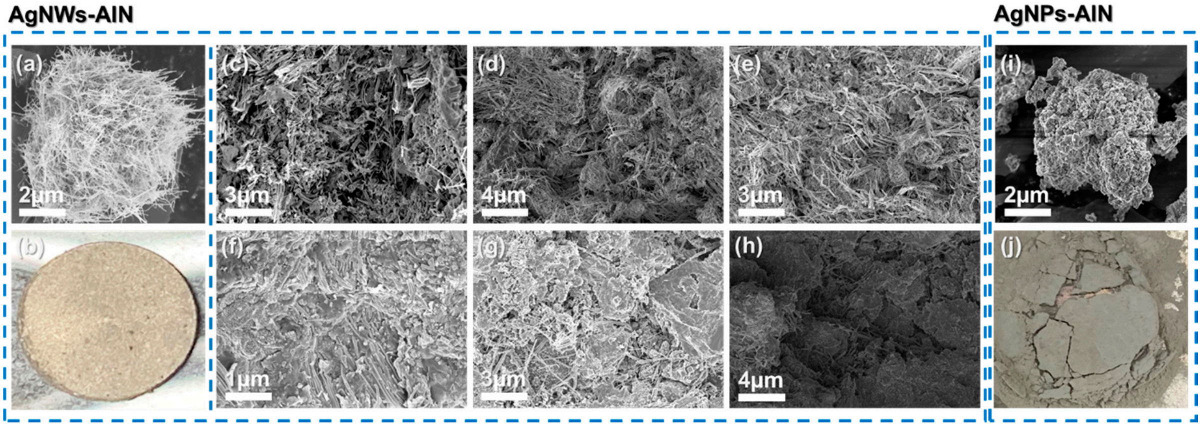

Figure S2. FE-SEM images of (**a**) AA filler and (**b**) 3D network by sintered AA fillers. Cross-sectional images of 3D network ((**c**) 30s, (**d**)1 min, and (**e**) 2 min and composites ((**f**) 30 s, (**g**) 1 min, and (**h**) 2 min) according to hot-pressing time. Images of (**i**) AgNPs-AlN and corresponding (h) network by sintered AgNPs-AlN.

Table S1. Comparison of composites thickness according to filler ratio.

Composites	AlN-to-AgNW Weight Ratio	Thickness (mm)
400-1:2	1:2	1.243
400-1:1	1:1	1.551
400-2:1	2:1	1.856
400-3:1	3:1	2.08
